# Insights on the Role of PGRMC1 in Mitotic and Meiotic Cell Division

**DOI:** 10.3390/cancers14235755

**Published:** 2022-11-23

**Authors:** Valentina Lodde, Rodrigo Garcia Barros, Laura Terzaghi, Federica Franciosi, Alberto Maria Luciano

**Affiliations:** Reproductive and Developmental Biology Laboratory, Department of Veterinary Medicine and Animal Sciences, Università degli Studi di Milano, 20122 Milano, Italy

**Keywords:** progesterone receptor membrane component 1, spindle, cytokinesis, cell division, cytoskeleton, mitosis, meiosis

## Abstract

**Simple Summary:**

PGRMC1 is a multifunctional protein regulating multiple aspects of cell proliferation, including cell viability, apoptosis, entry into the cell cycle, and the subsequent progression of cell division. In this review, we highlight the emerging role of PGRMC1 during the M phase and cytokinesis, which, compared to other PGRMC1 functions, have received modest attention from the scientific community. Mechanistically, we present insights into how the association with the chromosomal passenger complex and the cytoskeleton and their participation in membrane trafficking might contribute to the control of cell division.

**Abstract:**

During mitosis, chromosome missegregation and cytokinesis defects have been recognized as hallmarks of cancer cells. Cytoskeletal elements composing the spindle and the contractile ring and their associated proteins play crucial roles in the faithful progression of mitotic cell division. The hypothesis that PGRMC1, most likely as a part of a yet-to-be-defined complex, is involved in the regulation of spindle function and, more broadly, the cytoskeletal machinery driving cell division is particularly appealing. Nevertheless, more than ten years after the preliminary observation that PGRMC1 changes its localization dynamically during meiotic and mitotic cell division, this field of research has remained a niche and needs to be fully explored. To encourage research in this fascinating field, in this review, we will recap the current knowledge on PGRMC1 function during mitotic and meiotic cell division, critically highlighting the strengths and limitations of the experimental approaches used so far. We will focus on known interacting partners as well as new putative associated proteins that have recently arisen in the literature and that might support current as well as new hypotheses of a role for PGRMC1 in specific spindle subcompartments, such as the centrosome, kinetochores, and the midzone/midbody.

## 1. Introduction

PGRMC1 is a small transmembrane protein that is highly conserved in eukaryotes and has been related to an unusually high number of cellular functions, some of which were discovered unexpectedly from the screening of different proteins in relevant biological models. The multiplicity of the PGRMC1 function has been extensively reviewed [1,2,3], which is also why this Special Issue has been proposed [1].

The primary goal of this review article is to highlight the current knowledge of PGRMC1 function in regulating mammalian cell proliferation, specifically by mechanisms set in motion during the progression of cell division (M phase/cytokinesis), which, so far, has received less attention from the scientific community. Although PGRMC1, together with its partner PGRMC2, participates in many other functions that impact cell proliferation, such as cell viability and apoptosis and entry into the cell cycle, these functions have been reviewed elsewhere and will not be further discussed here [3,4,5].

At first glance, the question is whether a membrane protein can interact and eventually modulate cytoskeletal elements. Indeed, many recent studies have revealed the complexity of cell division, which potentially holistically involves every part of the cell. The formulation of the hypothesis about how PGRMC1 is involved in cell division cannot be separated from a detailed description of the process itself. Therefore, in the first part of the review, we summarize the key mechanisms of cell division that might be important in the context of PGRMC1 biology: this part is mostly a summary of comprehensive reviews on specific aspects of cell division. The current literature revealing the PGRMC1 function in cell division is reviewed and critically discussed in the second part. Finally, future directions are suggested.

## 2. Cell Division

Mitosis and meiosis are two forms of cell division conserved in eukaryotes and evolved from a common ancestor by adapting to different selective pressures [6]. During mitosis, the spindle microtubules “capture” the mitotic chromosomes (each made of two identical sister chromatids) and move them to the equator (congression) so that, during metaphase, pairs of sister chromatids become bioriented. During anaphase, chromatid cohesion is lost, and the two sister chromatids are pulled apart to opposite sides of the cell (segregation), where they decondense and are re-surrounded by the nuclear envelope during telophase [7,8]. Conversely, during the first meiotic division, the spindle arranges pairs of homologous chromosomes (each consisting of pairs of sister chromatids) at the equator of the spindle. Therefore, homologous chromosomes are kept side by side on the spindle equator facing opposite poles in metaphase I, and each of them is pulled apart by spindle microtubules in the subsequent phases of meiosis I [7,9]. Subsequently, the segregation of sister chromatids, occurring during the second meiotic division, mirrors mitotic division [7].

Despite the highlighted differences, both types of cell division rely on the function of a cytoskeletal “nanomachinery” [7] made of (1) the microtubule-based spindle and its associated motor proteins that drive chromosome/chromatid segregation [8] and (2) the actin cytoskeleton, which exerts crucial roles during mitotic entry, throughout chromosome segregation and, later on, during cytokinesis, when the actin-based contractile ring drives cytoplasm division into daughter cells [10,11,12,13,14,15]. In addition, the kinetochore, made up of many proteins that associate with the centromeric region of mitotic chromosomes, forms the mechanical hub of chromosome attachment to the spindle, aiding in correct congression, orientation, and subsequent segregation [9,16,17]. The kinetochore also has a key role in preventing aneuploidy since it is implicated in the spindle assembly checkpoint (SAC, see below), which ensures that chromosome segregation starts only after the correct attachment of all kinetochores and microtubules is established [9,16,17].

Chromosome/chromatid segregation and cytokinesis are highly interconnected and coordinated processes in both types of cell division. For example, the spindle itself dictates the site of furrow ingression. In turn, the position of the spindle within the dividing cell determines the symmetry of cell division [18,19]. Thus, if the spindle is centrally located, each daughter cell will contain approximately half of the original cytoplasmic content. On the contrary, if the spindle is closer to one of the cell boundaries, the cytoplasm will be unevenly distributed into the daughter cells, resulting in an asymmetrical division. Notably, symmetry—or lack thereof—during cell division dictates the fate of the daughter cells in many cell types [12,19,20,21,22,23,24,25]. In mammalian oocytes, for instance, two consecutive asymmetrical divisions occur to generate a large cell (the ovum) and two small cells (the polar bodies), each containing half of the genetic material of the dividing cell and a minimal quantity of the oocyte cytoplasm. The two polar bodies degenerate, while the ovum continues its journey by combining its genetic material with the paternal material in a process called syngamy [26]. 

**Figure 1:**
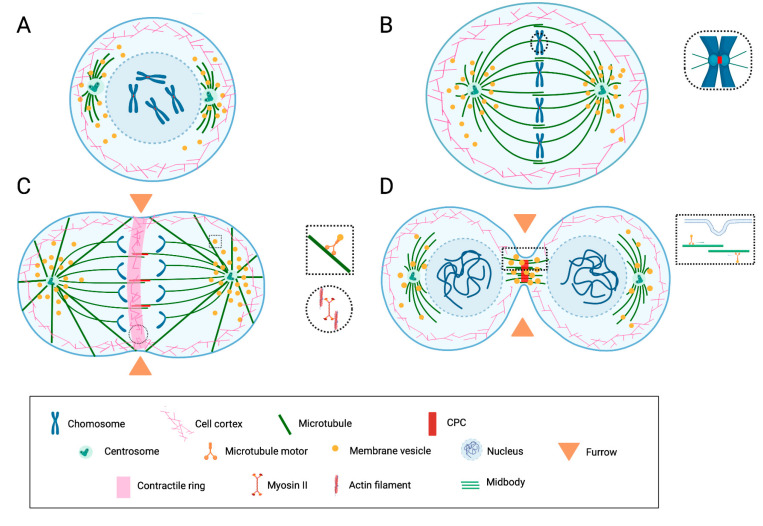
Schematic representation of the key features of mitotic cell division and cytokinesis. (**A**) During prophase/prometaphase, cell rounding occurs due to the rearrangement of the actin cytoskeleton. The duplicated centrosomes migrate around the nucleus, nucleate the spindle microtubules, and organize the spindle poles. The nuclear envelope breaks down, and the spindle microtubules promote mitotic chromosome congression. (**B**) During metaphase, the spindle consists of astral microtubules, which link spindle poles to the cell cortex; chromosomal and kinetochore microtubules, which overall link the chromosomes to poles; and interpolar microtubules, which link the two poles. The proteins of the chromosomal passenger complex (CPC) are concentrated at the centromere. (**C**) During anaphase, the chromatids segregate. The division plane is determined by a mechanism involving interactions between the cortex and the spindle. The cleavage furrow containing an actomyosin ring assembles and begins to contract. The CPC relocalizes at the central spindle. (**D**) During telophase/abscission, the nuclear envelopes reassemble around the decondensing chromosomes. The contractile ring contracts further, leading to the ingression of the furrow and constricting interpolar microtubules of the midzone into a restricted area (midbody). The CPC is concentrated in the midbody. During abscission, the furrow “seals” and divides the daughter cells via a mechanism thought to involve vesicle transport/exocytosis. Substantial rearrangements of the membranous compartment occur at all stages. Membrane vesicles are found associated with the spindle and participate in membrane remodeling during abscission. Image inspired by and modified from [7,31,33]. Created with BioRender.com (access on 10 November 2022).

The mechanisms by which actin filaments and microtubules generate forces that ultimately move cellular elements and serve as tracks for motor proteins have been the subject of intensive studies and are reviewed elsewhere [7,10,18,27,28,29,30,31,32,33]. Herein, a few general features of cell division are recapitulated to support the discussion about putative PGRMC1 functions in the second part of the paper. These features are summarized in Figure 1. 

### 2.1. The Coordinated Role of the Cytoskeleton

Cytoplasmic and nuclear events are highly interconnected and coordinated from the beginning of mitotic entry and spindle assembly [10]. The cellular architecture globally reorganizes, leading to so-called “mitotic cell rounding” [10,34]. In this stage, typical events include (1) the transient loss of cellular adhesion, which is, in turn, induced by focal adhesion disassembly [35,36,37], (2) the separation of centrosomes, which is powered by the combined action of motor proteins, microtubules, and actomyosin [38,39,40,41], (3) the assembly of the actomyosin cortex [42,43], and (4) the substantial remodeling of the nucleus [44,45].

The formation and proper function of the bipolar spindle are crucial for cell division. At least two mechanisms of bipolar spindle formation have been described. In the first one, which is generally active in mitosis, the microtubules nucleate from the centrosomes [8,46,47,48]. In the second one, which is mainly described in oocytes, centrosomes are lacking, and the chromosomes induce microtubule assembly [7,8,49]. Within this modality, however, species-specific differences have also been described [50,51,52] (see Figure 2 in [51]); for instance, in mouse oocytes, canonical centrosomes are functionally replaced by acentriolar microtubule-organizing centers (MTOCs) [50], while human, bovine, and porcine oocytes lack distinct acentriolar MTOC foci at their spindle poles [53,54]. These and other differences in the localization of spindle-related structures might indicate the species-specific functions of some proteins during oocyte meiosis.

Signaling that leads to cytokinesis commences as early as anaphase when the microtubules drive chromatid segregation to the opposite poles of the spindle [31,32]. In this stage, the overlapping microtubules in the midzone form the central spindle, and RhoA-GTP-dependent signaling between the central spindle and the cell cortex is established [55]. This spindle/cortex cross-talk is crucial for the formation of the actomyosin-based contractile ring, which, in turn, drives cytokinesis [32]. As the contractile ring constricts, the spindle midzone matures to form a compact structure called the “midbody”, which ultimately organizes the intercellular bridge and drives the final abscission [32].

### 2.2. The Spindle Assembly Checkpoint (SAC) and the Chromosomal Passenger Complex (CPC)

In metazoans, the SAC is an essential pathway that prevents chromosome missegregation and aneuploidy. Many proteins and protein complexes centered in the kinetochore during prometaphase/metaphase contribute to its integrity [9,16,17]. The SAC ultimately targets the Anaphase-Promoting Complex (APC) by preventing the destruction of cyclin B and securin, thus keeping sister chromatids together. Only when all chromosomes become bioriented and the SAC senses the proper chromosome–microtubule tension is the checkpoint system finally turned off. Then, a cascade of reactions leads to the exit from mitotic arrest, the activation of the APC, the loss of sister chromatid cohesion, and anaphase progression [9,16,17].

A key player of the SAC is the mitotic checkpoint complex (MCC), which contains the APC activator CDC20 and the spindle assembly checkpoint proteins MAD2, BUBR1/Mad3, and BUB3. Other “core” SAC components include the kinase Aurora-B (AURKB). These proteins are required to amplify the SAC signal and the rate of MCC formation (reviewed in [16]).

A mechanism similar to the SAC has been found in meiosis, and it involves the same crucial components [9,56,57]. However, during meiosis I, sister chromatid cohesion is partially retained to allow homologous chromosome segregation, implying the involvement of meiosis-specific components and tight regulation by kinase and phosphatase enzymes [9]. 

In addition to being part of the SAC, AURKB is the key component of one crucial “conductor” of cell division: the chromosomal passenger complex (CPC), which also comprises the inner centromere protein INCENP, survivin, and borealin [30,33,58,59,60,61]. The CPC changes its localization dynamically during mitotic and meiotic cell division: it associates with the chromatin in prophase, the centromeres in metaphase and early anaphase, and the midzone and midbody in late anaphase and telophase, respectively [30,33]. The dynamic localization correlates with its multiple functions, which include the formation of the bipolar spindle and its stability, the centromeric cohesion and regulation of kinetochore-microtubule attachments, the correct alignment of chromosomes on the spindle equator and the SAC, the formation of the central spindle in anaphase, and, later on, the completion of cytokinesis [30,33].

### 2.3. The Role of the Membranous Compartment

In addition to the cytoskeletal elements and associated motor proteins, all components of the cytoplasm, including the organelles of the endomembranous system, are substantially rearranged during both mitotic and meiotic cell division, contributing to their successful completion [62,63,64,65,66,67,68].

For instance, the endoplasmic reticulum (ER) changes its localization and structure as soon as the cell enters mitosis. When the nuclear envelope breaks down, the ER changes from its characteristic interphasic morphology to extended cisternae, with a reduction in tubule structures [69,70]. As mitosis progresses, the ER accumulates and aligns along the mitotic spindle, at the spindle poles, and, in some organisms, along the central spindle/midbody [71,72]. In addition, ER tubules associate with chromatin during late mitosis, which is essential for nuclear envelope reformation and the organization of the nuclear pore complex [66].

While membranes largely surround the spindle apparatus and seem to be essential for spindle function [73], electron microscopy studies have revealed that within the spindle, there is a relatively low abundance of ER- and Golgi-derived membranes and coated vesicles [74,75,76,77]. Nevertheless, membrane trafficking has recently emerged as a key component of cell division. Strikingly, recent findings reveal that endocytic membrane trafficking is involved in trafficking to and from the centrosome [78,79]. At the centrosome, endocytic membrane trafficking has a role in centrosome maturation and duplication [79,80,81,82,83]. Interestingly, recycling endosomes have been found at the spindle poles, at the central spindle’s boundaries, and in the midbody’s proximity [84,85,86,87]. Functionally, at these locations, endosomes bearing distinct Rab proteins have been implicated in the formation, positioning, and organization of the mitotic spindle, the congression of chromosomes, and cytokinesis [82]. In cytokinesis, membrane trafficking is crucial during abscission, when distinct machinery involving the endosomal sorting complex required for transport III (ESCRT-III) splits the plasma membrane of the nascent daughter cells [31,32,64].

In the context of membrane trafficking regulating cell division, a special mention has to be reserved for clathrin and clathrin-coated vesicles, mainly because the perturbation of proteins involved in membrane dynamics, including clathrin and dynamin, disrupts cytokinesis [62,88,89,90,91,92]. Clathrin-coated structures change localization dynamically from the spindle to a region between the separating chromosomes and subsequently disappear in the equatorial region. It has been hypothesized that this directional movement drives crucial membrane remodeling and/or signaling processes during cell division [88].

Interestingly, clathrin also controls earlier stages of cell division, localizing to the mitotic spindle until telophase [93,94]. However, data suggest that spindle-associated clathrin is not bound to membranes, but it directly binds to the spindle through the amino-terminal domain of the clathrin heavy chain [94,95]. Importantly, clathrin provides a structural lattice that organizes and stabilizes the kinetochore microtubules at the spindle, thus exerting a role in chromosome congression and segregation [94,96,97]. Similar crucial functions for clathrin have been described in meiosis [98,99].

## 3. PGRMC1 and Cell Division

### 3.1. Experimental Evidence That PGRMC1 Participates in the Control of Cell Proliferation and Cell Division

The mechanism by which PGRMC1 controls cell division is difficult to dissect. In fact, the multiplicity and cell-specificity of PGRMC1 function [1,2,3,100] imply that interfering with its function likely affects key biological processes during interphase, in addition to cell division. In addition, although the small molecule AG205 has been used as a PGRMC1 inhibitor, it is becoming evident that its effect is far from specific [1,101,102]. Therefore, caution should be taken when interpreting findings derived solely from AG205 treatment, unless they are validated by other means. Unfortunately, no specific PGRMC1 chemical inhibitors have been identified to date. 

Under these circumstances, the most informative studies are those in which PGRMC1 expression is experimentally altered (Table 1). Notably, all of the studies cited in Table 1 directly measured cell proliferation, either by assessing mitotic/meiotic progression, the fold increase in cell number, and the tumor mass or by using the MTT assay, previously validated by cell counting [103,104,105].

In the following paragraphs, we will discuss what we have learned from these studies. Nevertheless, it should be highlighted that only a few of them experimentally tested the hypothesis that PGRMC1 is directly involved in the progression of cell division, while the majority concentrated on cell proliferation and the regulation of tumor growth, supporting a role in cell cycle regulation, either direct or indirect.

#### 3.1.1. Control of Mitotic Cell Division

The observation that PGRMC1 is overexpressed in many types of cancers was probably the first indication of a relationship between its expression and cell proliferation. After the discovery of PGRMC1 as a putative mediator of progesterone action [1,2,116], many studies demonstrated that experimentally downregulating PGRMC1 in somatic cells reduced cell growth. In these studies (Table 1), cancerous and non-cancerous cell lines where PGRMC1 was downregulated grew slower in vitro than their parental cells. Accordingly, using cell-line-derived xenograft models, several studies have demonstrated that tumors derived from PGRMC1-depleted cancer cells grow slower than those derived from their respective parental cell lines.

On the other hand, fewer studies have tested the hypothesis that the exogenous overexpression of PGRMC1 accelerates cell proliferation. In these studies, various breast cancer cell lines overexpressing PGRMC1 showed increased cell proliferation in response to various progestin treatments [103,104,105]. Importantly, PGRMC1 overexpression showed different outcomes that were both cell- and progestin-type-dependent.

Altogether, the studies listed in Table 1 led to the general conclusion that PGRMC1 promotes proliferation. However, this conclusion is rather simplistic since the differential regulation of the cell proliferation rate upon the attenuation or overexpression of a gene does not necessarily confirm its involvement in the regulation of cell division. Cell proliferation can, indeed, occur because of increased cell viability, which leads to reduced cell death in response to stressors or as the result of an increased frequency of mitotic cell divisions. In turn, cell division is controlled at different levels, including the entry into the cell cycle and progression throughout the mitotic phases. Most often, the balance of these events determines whether the proliferation of a group of cells will increase or decrease.

Notably, many studies have focused on the mechanisms by which PGRMC1 regulates cell viability and apoptosis [106,117,118,119,120,121]. In addition, some studies have found that PGRMC1, together with PGRMC2, the other member of the PGRMC family, participates in the entry into the cell cycle through a complex mechanism that ultimately involves the control of NFKB/p65 localization, thereby regulating its activity as a transcription factor [115]. Notably, from a mechanistic point of view, these studies focused on the entry into the cell cycle, which was assessed by BrdU incorporation or the expression of the G1/S component of the FUCCI cell cycle sensor [111,115]. Thus, a specific role in the progression of mitotic cell division was not directly assessed. Nevertheless, as also discussed below, it is important to note that, although interfering with PGRMC 1 and 2 expression/interaction increased the rate of entry into the cell cycle and increased the percentage of mitotic figures, this was not accompanied by increased cell proliferation, but rather cell death [111,115]. These studies and their relevance in reproductive cancer biology have been recently reviewed [3,4,5]. 

As anticipated, despite the clear experimental evidence that PGRMC1 downregulation slows cell proliferation, few studies have specifically focused on its function in cells undergoing mitotic cell division. For instance, to date, no published research has used synchronized cells, which makes the interpretation of the results difficult and probably leads to underestimating the precise role of PGRMC1 in mitotic progression. 

However, some experiments were conducted in our lab to test the hypothesis that PGRMC1 is directly involved in the progression of mitotic cell division. In these studies, siRNA-mediated PGRMC1 downregulation reduced cell proliferation in the primary culture of bovine granulosa cells (bGCs) [113]. Furthermore, the lower rate of cell proliferation was accompanied by an increase in cells in the G2/M phase of the cell cycle, which is consistent with an arrested or prolonged M phase. This observation was supported by time-lapse imaging revealing defects in mitotic progression when PGRMC1 was silenced, specifically during late karyokinesis [113]. These data confirmed previous studies in rat spontaneously immortalized granulosa cells (SIGCs), in which the transfection of an anti-PGRMC1 antibody slowed cell proliferation while increasing the percentage of mitotic figures [110]. Furthermore, in SIGCs, the depletion of PGRMC1 and its partner PGRMC2 increased the rate of entry into the cell cycle and the accumulation of cells in the metaphase stage that finally underwent cell death [111]. More recently, these observations were confirmed in conditional knockout mice in which *Pgrmc1* was depleted in the reproductive tract [115]. In this model, *Pgrmc1* KO was associated with an increased rate at which granulosa cells entered the cell cycle and, at the same time, with a ≥2-fold increase in follicular atresia [115].

Altogether, these data support the hypothesis that the downregulation of PGRMC1 would facilitate the so-called “mitotic catastrophe”, which is defined as cell death resulting from aberrant mitosis or, more precisely, the “atypical mechanism that senses mitotic failure and respond to it by driving the cell to an irreversible fate, be it apoptosis, necrosis or senescence” [122,123,124]. This hypothesis is relevant to cancer biology, as it would imply that PGRMC1 overexpression in cancerous cells sustains the propagation of abnormal cancer cells, helping them to escape mitotic catastrophe.

#### 3.1.2. Control of Meiotic Cell Division

To the best of our knowledge, only our group has used siRNA technology to downregulate PGRMC1 expression in mammalian oocytes. siRNA-mediated gene silencing is challenging in oocytes undergoing meiotic maturation (i.e., the transition from prophase I to metaphase II), because oocytes are transcriptionally silent at this stage. Nevertheless, this model is highly informative, as it allows PGRMC1 to be interfered with only at a precise phase of meiotic progression. In turn, this would exclude possible biases due to a reduced availability for other key processes in other stages of the cell cycle. Such a scenario is almost impossible to obtain in somatic cells undergoing mitosis. In our studies, using the bovine model, we were able to suppress mRNA and protein expression by approximately 40%, which was consistent with a proportional decrease in oocytes properly completing the first meiotic division by emitting the first polar body. In parallel, we observed an increased percentage of oocytes showing aberrant meiotic figures, with misaligned chromosomes on the metaphase II plate and, often, scattered chromosomes in the cytoplasm. The effect of PGRMC1 silencing by siRNA technology mirrored the effect of treating oocytes with increasing doses of AG205 [113]. Likewise, similar outcomes were obtained by perturbing PGRMC1 function by means of an anti-PGRMC1 injection [108]. Notably, the effect on meiotic progression was more robust when oocytes were treated with either AG205 or the blocking antibody, which could be due to a more immediate and stronger perturbance of PGRMC1 function. Nevertheless, we cannot exclude that both AG205 and the antibody could have interfered with some nonspecific (unknown) target that contributed to defective meiotic progression. Clearly, transgenic mouse models in which *Pgrmc1* expression is conditionally depleted in oocytes are highly encouraged to add insights into this intricate issue.

Although this review focuses on mammals, it should be mentioned that the effect of silencing PGRMC1 expression by means of both morpholino and CRISPR/Cas9 technology was tested in zebrafish oocytes [125,126,127]. Nevertheless, in teleost fish, as in amphibians, oocyte maturation is induced by a maturation-inducing steroid. This factor is secreted by ovarian follicular cells in response to a gonadotropin surge and initiates oocyte maturation by binding to a specific progestin receptor on the oocyte plasma membrane [128,129]. Accordingly, the above-mentioned studies focused on the putative participation of PGRMC1 in the process by which membrane progestin receptors induce oocyte maturation, rather than on a possible role in mediating the subsequent meiotic progression. 

#### 3.1.3. Putative PGRMC1 Mechanisms of Action Controlling the Progression of Mitotic and Meiotic Cell Division

Only a few hypotheses on the possible mechanisms by which PGRMC1 regulates mitotic/meiotic progression have been experimentally tested. These hypotheses include the interaction with key components of the CPC and the modulation of the stability of the spindle via interaction with tubulin [108,110,113,130,131,132,133]. These studies were restricted to ovarian cells, while one study used adrenocortical NCI-H295R cells [133].

Chronologically, our interest in PGRMC1 biology was triggered by the preliminary observation of its peculiar localization in maturing bovine oocytes (see below). To the best of our knowledge, no data have been published on maturing oocytes of other mammals. However, since some differences exist in the modalities by which the spindle is formed in mammals (see Section 2.1), it would not be surprising if differences in the localization of PGRMC1 were found. This would also imply that PGRMC1 subcompartmentalization might depend on interacting with other spindle proteins. Later, localization in the spindle apparatus was confirmed in somatic cells undergoing mitotic cell division.

The presence of PGRMC1 in the spindle apparatus is also confirmed by some proteomic studies that analyzed the composition of the mitotic spindle and by pull-down experiments of key proteins regulating mitotic progression (Table 2). Despite the differences in the methodological approaches used in these studies and possible issues related to specificity, it is evident that PGRMC1 was not consistently detected in all of the reported studies. This inconsistency may be due to cell-type specificity and, even more, to the stability of the spindle in different cell types. Likewise, it is possible that weak interactions are responsible for the maintenance of PGRMC1 at this location. As such, we cannot exclude that the preparation of the cellular fraction to be analyzed strongly impacts the presence of PGRMC1 at the spindle apparatus. In our work, for instance, PGRMC1 localization at the meiotic spindle was less evident when high doses of detergent were used in the permeabilization phase (unpublished data). 

One of the most remarkable, although not surprising, observations of studies focusing on cell division is the conserved action that PGRMC1 seems to exert in the late stages of mitotic division and oocyte meiosis, i.e., karyo-/cytokinesis. This function is consistent with the localization in the spindle apparatus in both cell types and with the colocalization with AURKB and A [108,110,113,130,131,132,133]. Specifically, immunofluorescence studies have shown that PGRMC1 changes its localization dynamically: in somatic cells, it associates with the spindle in metaphase, while it localizes to the midzone and the midbody in anaphase and telophase/cytokinesis [108,110,113,130,131,132,133]. At all of these stages, PGRMC1 was found in close proximity to AURKB by means of PLA technology. This finding suggests that PGRMC1 might mediate the action of the CPC complex, particularly at the midbody, where the interaction is most prominent [113]. This observation is relevant since events occurring at the central spindle are crucial for proper cell division [13,15]. 

In maturing bovine oocytes, in addition to the localization seen at the midzone and midbody, well-defined localization at the centromeres was also observed [108,113,130,132]. Further experimental evidence has shown that (1) PGRMC1 predominantly colocalizes with the active phosphorylated form of AURKB in maturing oocytes [108]; (2) PGRMC1 and AURKB are both mislocalized in oocytes with a high incidence of aneuploidy [130]; (3) altering AURKB function by using the AURKB inhibitor ZM447439 alters PGRMC1 localization in bovine oocytes, which is associated with meiotic defects [130]; (4) in adrenal mitotic NCI-H295R cells, PGRMC1, together with its partner PGRMC2, colocalizes with ALADIN, a nucleoporin that plays crucial roles in mitotic division by regulating AURKA; and (5) PGRMC1 was found among the proteins that associate with core SAC components, such as members of the BUB family [149]. Altogether, these findings support the hypothesis that PGRMC1 is part of a complex that mediates the function of the CPC complex and/or the SAC. Clearly, much more investigation is needed to confirm this hypothesis and finally reveal mechanistic insights. 

Part of the mechanism by which PGRMC1 controls cell division is related to its association with beta-tubulin, as demonstrated in in vitro studies using SIGCs and SKOV-3 cells [110]. Specifically, in SKOV3, the downregulation of PGRMC1 increased the stability of spindle microtubules, as assessed by the rate of beta-tubulin disassembly in response to cooling. In addition, the same study provided some insights into the possible role of the P4-PGRMC1 interaction in controlling the spindle function. In fact, the effect of PGRMC1 depletion was mirrored by treatment with a high dose of P4 (4 µM) in the parental SKOV-3 cells. However, when the same treatment was applied to PGRMC1-depleted cells, P4 could not increase microtubule stability over that observed in PGRMC1-depleted cells. These results indicate that the P4 effect on spindle microtubule stability is, at least in part, mediated by PGRMC1 or, conversely, that PGRMC1’s action on the spindle can be modulated by P4 [110]. 

Interestingly, the effect of P4 on microtubule assembly and its possible interaction with the microtubule-associated protein MAP2 were reported years ago in neuronal cells [151,152]. More recently, studies in zebrafish during gastrulation revealed that P4 modulates microtubule dynamics [153] by positively affecting microtubule plus-end growth and tracking straightness in large yolk cells. Although the authors did not experimentally test the participation of PGRMC1, they speculated that P4’s action on the microtubule is mediated by a non-genomic mechanism at this stage of gastrulation, suggesting PGRMC1 as a possible mediator [153].

## 4. Other PGRMC1 Functions Found in Interphasic Cells Providing Insights into How PGRMC1 Might Participate in the Progression of Cell Division

### 4.1. Interaction with the Actin Cytoskeleton and Function Mediating Cell Shape and Migration

In addition to its association with spindle microtubules, recent findings indicate that PGRMC1 interacts with key components of the actin cytoskeleton [102]. As discussed below, this evidence is particularly relevant in interphasic cells, where this interaction seems to be responsible for the emerging role of PGRMC1 in mediating cell shape and migration [154,155,156,157,158,159].

Between 2017 and 2020, Salsano and collaborators assessed the possible function of PGRMC1 in decidualization, in which cells typically change their shape, becoming rounded [158,159]. Using human endometrial stromal cells (ESCs) as an in vitro model, they revealed that (1) PGRMC1 changes its localization during decidualization and (2) that exogenous PGRMC1 overexpression inhibits in vitro decidualization, altering cytoskeleton rearrangement and prolactin secretion [158]. Further co-immunoprecipitation studies revealed a change in PGRMC1-associated proteins before and after decidualization. Strikingly, the vast majority of these proteins were implicated in endomembrane trafficking/cytoskeleton or mitochondrial function [159,160].

In 2020, Huang and collaborators used two oral squamous carcinoma cell lines with different metastatic potentials to examine invasion mechanisms [155]. Their comparative proteomic approach identified PGRMC1 as one of the targets involved in the invasion mechanism. The silencing of PGRMC1 by siRNA reduced invasion in vitro as well as in vivo using xenograft models [155]. Furthermore, in vitro, the phenotype was correlated with reduced activity and/or the expression of key components of the migration process [155]. Accordingly, Lee and collaborators [156] used a transgenic mouse model that spontaneously developed breast tumors, which were in turn backcrossed with Pgrmc1 knockout (KO) mice to demonstrate that the tumors that developed in the *Pgrmc1* KO mice had a lower metastatic ability and a lower expression of focal adhesion kinase (FAK) [156]. This evidence was confirmed in in vitro studies with PGRMC1-depleted MCF-7 and MDA-MB-231 breast tumor cells, which had lower in vitro migratory activity than the parental cell lines [156]. 

In the same years, Thejer and collaborators [154] discovered that PGRMC1 phosphorylation influences the cell shape, motility, and invasion of pancreatic cancer cells (MIA PaCa-2, MP). In this study, MP cells were stably transfected with wild-type HA-tagged PGRMC1 or several phosphorylation mutants. Interestingly, cells transfected with wild-type PGRMC1 mainly exhibited an elongated morphology, similar to the parental cell lines. In contrast, the ones with PRGMC1 mutants showed a rounded morphology, which was dependent upon activated Rho-associated protein kinase (ROCK), which controls the stiffening of cortical actomyosin [154]. In addition, proteomic analysis of these cells showed that the increased motility of cells expressing PGRMC1 mutants was associated with a higher abundance of proteins of the actin cytoskeleton [154]. 

Strikingly, the same group conducted pull-down experiments in the same MP cells, revealing that many proteins of the actin cytoskeleton associate with HA-tagged PGRMC1 [102]. In addition, it has been reported that the cytochrome b5 domain found in PGRMC1 contains a region of predicted coiled-coil formation similar to that of several myosins [161]. 

**Figure 2:**
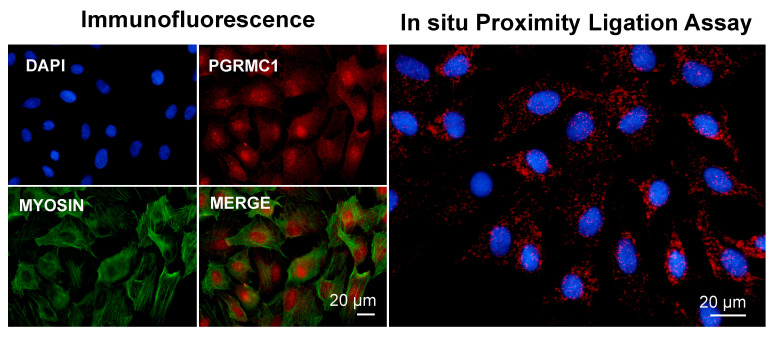
Images showing immunofluorescence and in situ proximity ligation assay (PLA) to assess the association of PGRMC1 and myosin in in vitro cultured bovine granulosa cells. Analysis was conducted as described in [113,163] with proper combination of antibodies, which were rabbit anti-PGRMC1 (Sigma Aldric Prestige antibody HPA002877, 1:50) and mouse monoclonal non-muscle myosin IIA antibody (2B3) (Novus Biological H00004627-M03, 1:100). Nuclei were counterstained with DAPI. Data were presented at the 51st Annual Meeting of the Society for the Study of Reproduction, 10–13 July 2018, New Orleans, Louisiana, USA, and the 2018 Gordon Research Conference in Mammalian Reproduction, 29 July–3 August 2018, Barga, Lucca, IT.

Altogether, the above findings correlate well with a putative PGRMC1 role in participating in actin-/myosin-mediated cellular function, such as contraction, which, mechanistically, would be mediated by myosin-interacting proteins [154,161]. In our laboratory, we conducted preliminary experiments to study the PGRMC1-myosin II association by means of immunofluorescence and an in situ proximity ligation assay (PLA), an immune-based technique that reveals whether two proteins are in intimate proximity [162]. Although it is possible that some nonspecific signal was generated, and more corroborating experiments are needed to prove a direct interaction, our results seem to support the hypothesis that PGRMC1 participates in actin-/myosin-mediated cellular function (Figure 2). 

Most likely, interaction with the components of the actin cytoskeleton is retained in mitosis, which could have profound implications for cell division, as outlined in the first part of this review. The rounded phenotype observed in MP cells bearing a PGRMC1 mutation [154] and in ESCs undergoing decidualization [159] is particularly relevant in the context of the role of PGRMC1 in controlling cell division, given that cell rounding also characterizes entry into mitosis, as outlined in Section 2.1 [10,34]. 

In addition, the hypothesis that PGRMC1 participates in the control of myosin-mediated actin contraction is consistent with our studies showing that PGRMC1 depletion causes karyo-/cytokinesis defects in both mitosis and meiosis, as the contraction of the contractile ring, which is initiated by signals emanating from the central spindle in anaphase, is central to proper cell division. We hope that this assumption will soon be tested. In this regard, co-immunoprecipitation studies [102,159] provide some insight into the possible molecular mechanisms of PGRMC1 function. A comparison of immunoprecipitated proteins in synchronized mitotic versus interphasic cells would broadly advance this field of research.

Finally, another exciting aspect that deserves attention is the emerging evidence that P4 regulates the actin cytoskeleton in several cell types [164,165,166,167], which could be mediated by PGRMC1 in both interphasic cells and cells undergoing cell division.

### 4.2. Control of Membrane Trafficking

Another known PGRMC1 function that likely impacts cell division is the control of membrane trafficking [2,100,168,169,170], which, indeed, has pivotal functions during mitosis (see Section 2.3). 

As anticipated in the previous paragraph, PGRMC1 pull-down experiments were recently conducted in human ESCs to investigate the possible implication of PGRMC1 in the process of decidualization [159,160]. Notably, most of the PGRMC1-coprecipitated proteins were involved in endomembrane trafficking/cytoskeleton or mitochondrial functions, which links PGRMC1 to membrane trafficking, cytoskeletal remodeling, and cell shape. 

Endocytosis substantially contributes to the remodeling of cellular membranes, and one of the players in endocytic membrane trafficking is clathrin [171]. Recently, Riad and collaborators found that PGRMC1 and the Sigma-2 Receptor/TMEM97 form a complex that mediates LDL internalization by the LDL Receptor in HeLa cells [172]. Specifically, the authors reported LDLR internalization via clathrin-mediated endocytosis in a mechanism requiring PGRMC1 and TMEM97, as knockout of PGRMC1 or TMEM97 attenuated LDLR endocytosis. Nevertheless, knockout of TMEM97 and/or PGRMC1 did not affect somatostatin and insulin uptake, which are also clathrin-dependent [172]. Therefore, the PGRMC1-clathrin association seems to affect a subset of clathrin-mediated endocytosis.

**Figure 3:**
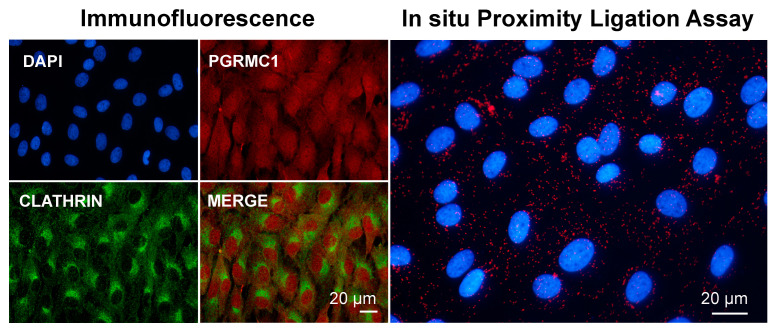
Images showing immunofluorescent and in situ proximity ligation assay to assess the association of PGRMC1 and clathrin in in vitro cultured bovine granulosa cells. Analysis was conducted as described in [113,163] with proper combination of antibodies, which were rabbit anti-PGRMC1 (Sigma Aldric Prestige antibody HPA002877, 1:50) and mouse monoclonal anti-clathrin heavy-chain antibody (X22) (ThermoFisher Scientific, 1:500). Nuclei were counterstained with DAPI. Data were presented at the 48th Annual Meeting of the Society for the Study of Reproduction, 18–22 June 2015, San Juan, Puerto Rico, USA.

Preliminary experiments conducted in our laboratory by means of immunofluorescence and PLA seem to confirm that at least part of PGRMC1 present in the cell cytoplasm colocalizes and associates with clathrin (Figure 3, Lodde and Terzaghi, unpublished). This is consistent with a model of clathrin-mediated endocytosis of the LDLR via a mechanism requiring PGRMC1 and TMEM97, as proposed by Riad and collaborators [172]. As in the case of the association with myosin shown in Figure 2, more experimental evidence is needed to confirm this interaction. To the best of our knowledge, studies on a possible interaction/mechanism of action involving PGRMC1 and other mediators of membrane trafficking are lacking. Research in this area is foreseen, as endocytosis plays an important role in cell division [173].

As described in Section 2.3, in addition to its role in mediating endocytosis, clathrin exerts a peculiar function during mitotic and meiotic cell division [174]. Interestingly, Rao and collaborators analyzed the composition of the spindle proteome and phosphoproteome in cells that were depleted of clathrin, with the aim of describing the clathrin-dependent mitotic spindle proteome [147]. In this study, PGRMC1 was detected in as many as four out of five biological replicates of the phosphoproteome but in only two out of five biological replicates of the spindle proteome. A similar situation was reported for the well-known spindle protein TACC3. Unfortunately, PGRMC1, as well as TACC3, was not further considered in the study. Nevertheless, this result raises the intriguing question as to whether PGRMC1’s presence/function in the spindle is somehow related to clathrin.

**Figure 4:**
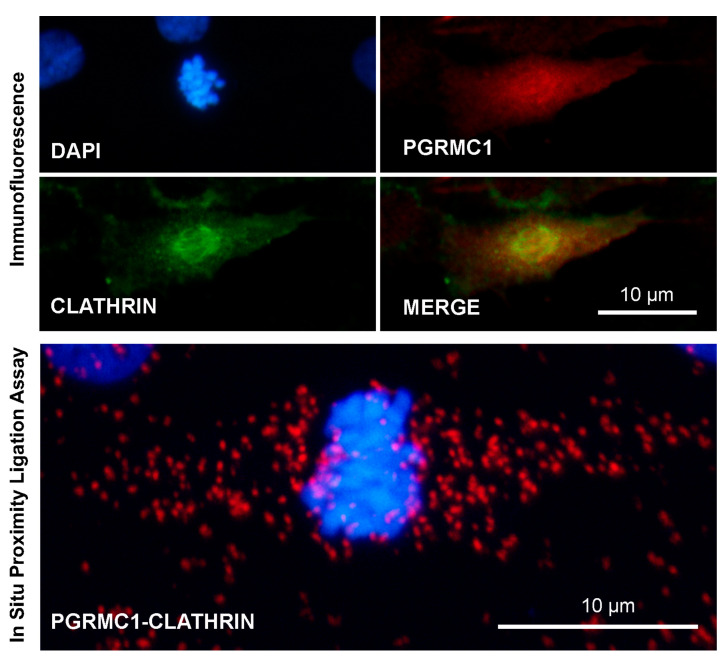
Images showing immunofluorescent and in situ proximity ligation assay to assess the association of PGRMC1 and clathrin in in vitro cultured bovine granulosa cells undergoing mitotic division. Analysis was conducted as described in [113,163] with proper combination of antibodies, which were rabbit anti-PGRMC1 (Sigma Aldric Prestige antibody HPA002877, 1:50) and mouse monoclonal anti-clathrin heavy-chain antibody (X22) (ThermoFisher Scientific, 1:500). Nuclei were counterstained with DAPI. Controls were performed by eliminating one of the two primary antibodies, as shown in Supplementary Figure S1. Data were presented at the 48th Annual Meeting of the Society for the Study of Reproduction, 18–22 June 2015, San Juan, Puerto Rico, USA.

A preliminary observation in our laboratory confirmed, as expected, that both PGRMC1 and clathrin localize in the cytoplasm and in the spindle area of mitotic bGCs (Figure 4, Lodde and Terzaghi, unpublished). Furthermore, these two proteins seem to be in close proximity in mitotic cells, as assessed by PLA. Nevertheless, this putative association does not seem to be restricted to the spindle but rather seems to be spread throughout the whole cell. It could be that PGRMC1 can associate (directly or indirectly) with clathrin in both its forms, the vesicle-associated and non-associated ones. Clearly, no conclusion can be drawn from this sole observation, but we believe that it can stimulate further research on this intriguing aspect of PGRMC1 biology. For instance, a key concept of clathrin biology is its inability to directly bind to membranes or cargo; instead, clathrin binds to adaptor proteins, which in turn can bind to membranes or proteins destined for trafficking [95,175,176]. The temptation to hypothesize that PGRMC1 is part of this mechanism is strong. 

Finally, except for the study highlighting PGRMC2 (and thus PGRMC1) with the nuclear pore complex protein ALADIN [131,133], nothing else has been published about the possible implication of PGRMC1 localization at the nuclear envelope. This aspect might also be relevant, as the remodeling of the nuclear envelope is primarily implicated in cell division. To date, this issue is entirely unexplored. Similarly, the function of the fraction of PGRMC1 that is retained in the ER during mitosis, if any, is unknown.

Clearly, more investigation is needed. An exciting hypothesis worth testing would be that the fraction of PGRMC1 associated with intracellular vesicles also participates in the trafficking of these vesicles along the cytoskeleton. This might regulate the function of other cargo proteins. On the other hand, vesicle trafficking could also bring PGRMC1 into different endomembranous compartments, where it could interact with different effector proteins as it exerts its many functions. Such a mechanism could be relevant in both interphase and mitosis/meiosis and could simultaneously explain how PGRMC1 might associate with the spindle. In this view, it would be interesting to test whether PGRMC1 mediates trafficking to the centrosome or the central spindle in both mitotic and meiotic cell division. In addition, in oocytes, it would be interesting to test whether PGRMC1 participates in the mechanisms by which the cooperative action of vesicles and actin filaments promote the movement of the spindle (see Section 2.1).

## 5. Conclusions and Future Directions

As with every complex matter, PGRMC1 is undoubtedly fascinating, and the answers to the many still-unresolved questions related to its biology will come only after extensive research. One of these intriguing questions relates to the difference, if any, in PGRMC1 function in cancerous and non-cancerous cells. As outlined in this review, the information we have acquired so far suggests that part of the mechanism by which PGRMC1 regulates cell function relates to its ability to interact with the cytoskeleton and probably, directly or indirectly, with other cytoskeleton-related regulatory molecules. This function, which modulates cellular function in interphasic cells, is retained during cell division, conferring PGRMC1 with additional properties. 

Much essential information will have to be experimentally acquired, and the primary purpose of this review is to stimulate new research in this field. First, we need to clarify the “origin” of the PGRMC1 associated with the mitotic and meiotic spindles. Is it membrane-bound? Does it come from the membranous compartment? Or rather, does it come from the nucleus? In this view, PGRMC1, which also has nuclear and nucleolar localization [108,118,163], could act like many other nuclear/nucleolar proteins that shuffle from the nucleus to the mitotic/meiotic chromosomes, such as NumA, nucleolin, or AURKs themselves [177,178]. Further, it is pivotal to understand which domain of the protein is responsible for PGRMC1 localizing at the spindle and whether dimerization and heme binding are part of this mechanism [114,179].

It will also be of primary importance to assess the extent to which translational regulation and/or post-translational modifications, which have been shown to be important in the regulation of PGRMC1 function, are involved in the mechanism that ensures proper localization/function of PGRMC1 during mitotic and meiotic division. In particular, PGRMC1 phosphorylation affects several biological processes, including cell shape and motility [154,180]. In addition, a phosphorylated form of PGRMC1 has been found among the phosphoproteins of the mitotic spindle in HeLa cells [136,147]. Ubiquitination and sumoylation have also been reported to modulate PGRMC1 localization and function; sumoylation, in particular, is important for nuclear localization [181,182,183].

Clearly, the precise mechanism of action by which the PGRMC1–cytoskeleton interaction affects cell division remains to be elucidated. The data in the literature clearly indicate that PGRMC1 cooperates with many other key proteins and complexes to exert its many functions (for example, see [125,181,184,185,186]). As hypothesized by many researchers, PGRMC1 could act as an adaptor protein in many different biological processes. Thus, PGRMC1’s action would depend on the proteins with which it interacts in different cellular and subcellular systems. In this view, knowing that PGRMC1′s interacting protein is of crucial importance, further studies are needed to deepen our knowledge on the role of PGRMC1/PGRMC2 functional interaction at the spindle [111,115,187]. Although the current proteomic studies can be used as initial data to reveal mechanistically relevant binding partners, pull-down studies should be performed on synchronized cells to more precisely reveal the mitotic-specific partners of PGRMC1.

Additional helpful information could also come from a phylogenetic analysis, which could, for example, reveal when the ability to bind the actin cytoskeleton arose during evolution. Similar studies have already been conducted to reveal crucial aspects of PGRMC1 biology [161,188].

We recognize that some of the hypotheses outlined in this review are probably too speculative, but we believe that the answers to the many questions related to PGRMC1 biology will only come if we think outside of the box. For example, Peluso and Pru recently suggested that PGRMC1 might be involved in the control of translation, given the high number of ribosomal proteins that co-immunoprecipitated with PGRMC1 [3,5]. Thus, what if all of the hypotheses are true at the same time? What if the known function of PGRMC1 is part of a more complex mechanism of action? What if PGRMC1 brings mRNA to specific subcellular sites and, in these sites, regulates their translation? Clearly, this story must continue.

## Figures and Tables

**Table 1:** Experimental evidence supporting a role for PGRMC1 in cell proliferation and cell division.

Reference Title	Year	Cell Type	Experimental Approach Used to Disturb PGRMC1 Function	Effect on Cell Proliferation (Phenotype)	Proposed Mechanism (If Any)	Ref.
Regulation of ovarian cancer cell viability and sensitivity to cisplatin by progesterone receptor membrane component 1	2008	Ovcar-3 cells	Overexpression of exogenous PGRMC1; siRNA-mediated gene silencing; transfection of antibody	Increased cell viability in response to cisplatin	Regulation of apoptosis and P4 antiapoptotic action	[106]
Progesterone receptor membrane component-1 regulates the development and Cisplatin sensitivity of human ovarian tumors in athymic nude mice	2009	Human ovarian cancer cells (SKOV-3 cells)	Gene silencing by short hairpin RNA knockdown approach; xenograft model of athymic nude mice	Lowered in vitro growth and reduced tumor xenograft growth	Regulation of apoptosis and P4 antiapoptotic action	[107]
Progesterone receptor membrane component 1 expression and putative function in bovine oocyte maturation, fertilization, and early embryonic development	2010	Bovine oocytes	Antibody injection	Impaired meiotic progression	Regulation of meiotic spindle function	[108]
Progesterone receptor membrane component 1 (Pgrmc1): a heme-1 domain protein that promotes tumorigenesis and is inhibited by a small molecule	2010	Human A549 non-small cell lung cancer cells and MDA- MB-468 breast cancer cells	siRNA-mediated gene silencing and short hairpin RNA knockdown approach; xenograft model of athymic nude mice	Reduced tumor xenograft growth	-	[109]
A novel role for progesterone and progesterone receptor membrane component 1 in regulating spindle microtubule stability during rat and human ovarian cell mitosis	2011	Rat spontaneously immortalized granulosa cells (SIGCs) and human ovarian cancer cells (SKOV-3 cells)	Antibody transfection; PGRMC1 downregulation	Lowered growth rate	Regulation of spindle function (microtubule-mediated process)	[110]
Progestogens and membrane-initiated effects on the proliferation of human breast cancer cells	2012	MCF-7	PGRMC1 overexpression	Increased cell proliferation in response to progestin treatment		[104]
Overexpression of progesterone receptor membrane component 1: possible mechanism for increased breast cancer risk with norethisterone in hormone therapy	2013	MCF-7	PGRMC1 overexpression; xenograft model of athymic nude mice	Increased cell proliferation of PGRMC1-overexpressing breast cancer cells in response to E2/NET combination		[103]
Progesterone receptor membrane component-1 (PGRMC1) and PGRMC-2 interact to suppress entry into the cell cycle in spontaneously immortalized rat granulosa cells	2014	Rat spontaneously immortalized granulosa cells (SIGCs)	siRNA-mediated gene silencing	Increased entry into the cell cycle without cell proliferation—PGRMC1- and/or PGRMC2-depleted cells accumulate in metaphase and undergo apoptosis	Regulation of entry into the G1 stage of the cell cycle through interaction with PGRMC2 and G3BP2	[111]
Progesterone receptor membrane component 1 promotes survival of human breast cancer cells and the growth of xenograft tumors	2016	MDA-MB-468 breast cancer cells	Short hairpin RNA knockdown approach; xenograft model of athymic nude mice	Reduced tumor xenograft growth	Regulation of cell viability	[112]
PGRMC1 participates in late events of bovine granulosa cells mitosis and oocyte meiosis	2016	Primary bovine granulosa cell culture; bovine oocytes	siRNA-mediated gene silencing	Decreased cell proliferation; accumulation of M-phase cells that eventually die; defective meiotic maturation	Regulation of cytokinesis and of mitotic spindle function through association with AURKB	[113]
Haem-dependent dimerization of PGRMC1/Sigma-2 receptor facilitates cancer proliferation and chemoresistance	2016	HCT116 cells and derived tumors in a model of liver metastases of human colon cancer	Stable PGRMC1 knockdown and xenograft model of NOG mice	Reduced cell proliferation of spheroids grown in vitro and reduced tumor growth in vivo	Regulation of EGFR and cytochrome P450 signaling	[114]
Progesterone receptor membrane components 1 and 2 regulate granulosa cell mitosis and survival through a NFKappaB-dependent mechanism	2019	Mouse ovarian cells	Conditional knockout PGRMC1 mice; siRNA-mediated gene silencing	Increased follicular atresia—ovarian granulosa cells of PGRMC1 conditional KO mice enter the cell cycle more frequently compared to controls but then do not seem to progress, causing increased follicular atresia	Regulation of entry into the cell cycle by an NFkB-mediated action	[115]
PGRMC1 Promotes Progestin-Dependent Proliferation of Breast Cancer Cells by Binding Prohibitins Resulting in Activation of ERalpha Signaling	2021	Various breast cancer cell lines	PGRMC1 overexpression	Increased cell proliferation in response to progestin treatment	Regulation of ERα signaling	[105]

**Table 2:** Proteomic studies revealing (or not) PGRMC1 association with the spindle apparatus.

Title	Year	Cell Type	Cellular Fraction	Method	Presence of PGRMC1 (Y/N)	Ref.
Dissection of the mammalian midbody proteome reveals conserved cytokinesis mechanisms	2004	Chinese hamster ovaries cells	Midbody	Mass spectrometry	N	[134]
Proteome analysis of the human mitotic spindle	2005	HeLa S3 cells	Mitotic spindle	Mass spectrometry	N	[135]
Phosphoproteome analysis of the human mitotic spindle	2006	HeLa S3 cells	Mitotic spindle	Mass spectrometry	Y	[136]
Molecular architecture of the kinetochore–microtubule interface	2008	Various cell type	Kinetochore	Review paper	N	[137]
Quantitative analysis of the human spindle phosphoproteome at distinct mitotic stages	2009	HeLa S3 cells	Mitotic spindle	SILAC technology	N	[138]
The protein composition of mitotic chromosomes determined using multiclassifier combinatorial proteomics	2010	Chicken DT40 cells	Mitotic chromosome (kinetochore)	SILAC technology	Y	[139]
Binding Partner Switching on Microtubules and Aurora-B in the Mitosis to Cytokinesis Transition	2010	HeLa S3 cells	M/C phase (microtubules)	SILAC MS	Y	[140]
Mitotic spindle proteomics in Chinese hamster ovary cells	2011	Chinese hamster ovaries cells	Mitotic spindle	Mass spectrometry	Y	[141]
Cell cortex composition and homeostasis resolved by integrating proteomics and quantitative imaging	2013	Human melanoma cells/HeLa cells	Cell cortex (MII cells)	LC-MS/MS	N	[142]
Cellular control of cortical actin nucleation	2014	Human melanoma cells/HeLa cells	Cell cortex (MII cells)	LC-MS/MS	N	[143]
A dynamic protein interaction landscape of the human centrosome-cilium interface	2015	293 T-REx cells	Centrioles	Mass spectrometry	Y	[144]
Whole-proteome genetic analysis of dependencies in assembly of a vertebrate kinetochore	2015	Chicken lymphoma B cell line DT40	Mitotic chromosome (kinetochore)	Mass spectrometry	N	[145]
Global phosphoproteomic mapping of early mitotic exit in human cells identifies novel substrate dephosphorylation motifs	2015	HeLa Cells	Mitotic spindle	SILAC technology	Y	[146]
The clathrin-dependent spindle proteome	2016	Human HeLa cells	Mitotic spindle (KT and centrosomes)	LC-MS/MS	Y	[147]
Spatial and proteomic profiling reveals centrosome-independent features of centriolar satellites	2019	Flp-In T-REx 293 (human)	Microtubules	mass spectrometry	N	[148]
Mapping Proximity Associations of Core Spindle Assembly Checkpoint Proteins	2021	HeLa Flp-In T-Rex and retinal pigment epithelium (RPE) cells	Kinetochore (BUB1 BUB1B BUB3) association	LC-MS/MS	Y	[149]
The Proteomic Landscape of Centromeric Chromatin Reveals an Essential Role for the Ctf19CCAN Complex in Meiotic Kinetochore Assembly	2021	Yeast (S cerevisiae)	Meiotic centromeres and kinetochores	Label-Free Mass Spectrometry (LFQMS)	N	[150]

## Supplementary Materials

The following supporting information can be downloaded at: https://www.mdpi.com/article/10.3390/cancers14235755/s1, Figure S1: Images showing controls omitting the primary antibodies of Immunofluorescent and In Situ Proximity ligation assay shown in Figure 2, to assess the association of PGRMC1 and Clathrin in in vitro cultured bovine Granulosa Cells undergoing mitotic division.
