# Supplementary material for: Insights on the Role of PGRMC1 in Mitotic and Meiotic Cell Division
Source: Cancers (Basel). 2022 Nov 23;14(23):5755. doi: 10.3390/cancers14235755 (PMC9736406; doi:10.3390/cancers14235755)
Supplement: Supplementary file 1 [file cancers-14-05755-s001.zip › cancers-1937533-supplementary.pdf]

cancers

MDPI

SUPPLEMENTARY FIGURE S1

Combination of antibodies

mouse anti Clathrin	+	-
rabbit anti PGRMC1	-	+
anti mouse A488 / PLA probe	+	+
anti rabbit TRITC / PLA probe	+	+

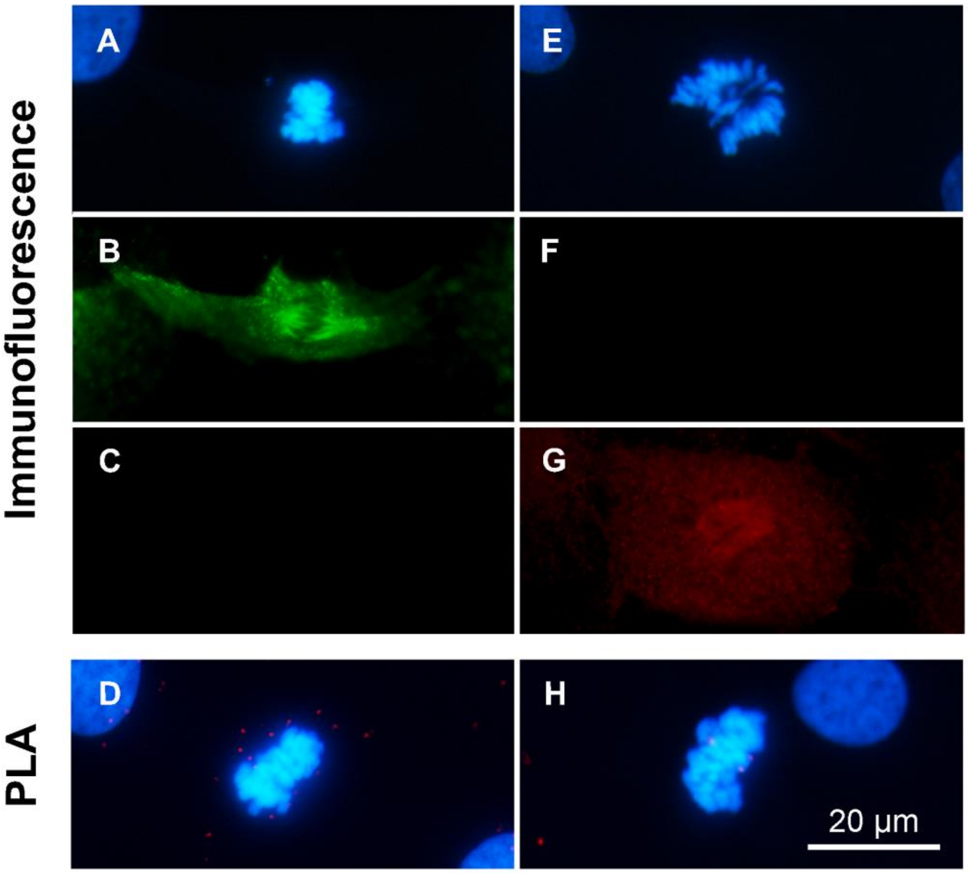

Supplementary Figure S1. Images showing controls omitting the primary antibodies of Immunofluorescent and *In Situ* Proximity ligation assay shown in Figure 2, to assess the association of PGRMC1 and Clathrin in *in vitro* cultured bovine Granulosa Cells undergoing mitotic division. Analysis were conducted as described in [113, 163] with proper combination of antibodies which were Rabbit anti PGRMC1 (Sigma Aldric Prestige antibody HPA002877, 1:50) and Mouse Monoclonal anti Clathrin, Heavy chain Antibody (X22) (ThermoFisher Scientific, 1:500). Nuclei were counterstained with DAPI. Controls were performed by eliminating one of the 2 primary antibodies. Both secondary antibodies in the immunofluorescent reactions, or the PLA probes, il the PLA were present. Data were presented at *48th Annual Meeting of the Society for the Study of Reproduction, 18-22 June 2015 San Juan, Puerto Rico, USA.*
